# Interplay between ROS and Autophagy in Cancer and Aging: From Molecular Mechanisms to Novel Therapeutic Approaches

**DOI:** 10.1155/2019/8794612

**Published:** 2019-08-04

**Authors:** Marco Cordani, Massimo Donadelli, Raffaele Strippoli, Alexandr V. Bazhin, Miguel Sánchez-Álvarez

**Affiliations:** ^1^Instituto Madrileño de Estudios Avanzados en Nanociencia (IMDEA Nanociencia), CNB-CSIC-IMDEA Nanociencia Associated Unit “Unidad de Nanobiotecnología”, Madrid 28049, Spain; ^2^Department of Neurosciences, Biomedicine and Movement Sciences, Section of Biochemistry, University of Verona, Verona, Italy; ^3^Department of Molecular Medicine Sapienza, University of Rome, Rome, Italy; ^4^Gene Expression Laboratory, National Institute for Infectious Diseases “Lazzaro Spallanzani” IRCCS, Rome, Italy; ^5^Department of General, Visceral and Transplantation Surgery, Ludwig-Maximilians University, Munich, Germany; ^6^Mechanoadaptation & Caveolae Biology Lab, Cell and Developmental Biology Area, Centro Nacional de Investigaciones Cardiovasculares (CNIC), Madrid 28029, Spain

Aging and cancer are highly related biological phenomena. Cellular processes that underpin several malignant phenotypic traits, including DNA damage responses, oxidative stress, metabolic rewiring, and cellular senescence, also contribute to aging. Reactive oxygen species (ROS) play an essential role as intra- and extracellular messengers, orchestrating functional and metabolic states of the cell through the regulation of different signaling pathways [1, 2]. Importantly, ROS levels are persistently elevated in cancer cells as a result of their increased metabolic activity, mitochondrial dysfunction, and activation of oncogenes [3]. However, ROS are also powerful oxidizing agents, which can induce cell injury upon modification of lipids, proteins, or DNA, altering normal cell physiology and increasing the risk of DNA mutation and tumorigenesis. Autophagy is a key node for the regulation of ROS levels as well as for ROS-dependent cellular regulation. Autophagy comprises salvaging processes, commonly triggered by metabolic stress responses by which macromolecules and organelles are targeted by autophagic vesicles to lysosomes for degradation and recycling of their constituents [4]. Many studies revealed that alterations in ROS and autophagy are implicated in cancer biology and aging. However, while it is established that high levels of ROS and impaired autophagy drive aging in mammalian cells, their role in regulating cancer cell death or survival is highly contextual and dependent on the source of stress, tumor particularities, and its metabolic status [5]. Despite the fact that both ROS and autophagy can promote tumorigenesis and tumor progression, their exacerbation may induce cell death following a nonspecific injury or an excessive degradation of macromolecules and cellular organelles required for cellular processes. Interestingly, many oncogenic stimuli that induce ROS generation also trigger autophagy, including nutrient starvation, mitochondrial dysfunction, and hypoxia, suggesting the existence of the interplay between ROS and autophagy. Among the plethora of signaling pathways regulating this interplay, the mechanistic Target Of Rapamycin Complex 1 (mTORC1) and 5′AMP-activated protein kinase (AMPK) interpret multiple cues, including oxidative stress, to integrate them with the control of energy management, anabolism, and cell growth [6]. Conversely, these signaling systems regulate metabolism and growth which are in turn the major ROS sources themselves. Thus, the understanding of the molecular mechanisms linking ROS and autophagy may acquire an exceptional significance to develop novel, tailored, preventive, and therapeutic strategies against cancer disease and aging processes.

In this special issue, we present seven research communications dedicated to the molecular mechanisms mediated by the crosstalk between ROS and autophagy involved in cancer development and progression and four review articles covering/providing a perspective of the state of the art on some of the current developments in this emerging field.

Melanoma is the most aggressive cancer with high mortality rate, especially when diagnosed late. Intriguingly, a number of studies support a key role of autophagy-related genes (ARGs) in melanoma onset. D. D'Arcangelo et al. demonstrated for the first time the expression levels of a large number of ARGs in several melanoma samples and identified three genes (BAG1, PEX3, and WIPI1) known to play a key role in autophagy, as novel relevant melanoma markers. Therefore, such molecules may represent valuable novel markers of melanoma onset and progression.

Different studies highlight the beneficial effects of exogenous preparations, such as plant-derived extracts to prevent cancer and aging through the regulation of the interplay between ROS and autophagy. Here, B. I. Fernandez-Gil et al. report novel molecular insights by which melatonin enhances the antitumor effects of irradiation and cisplatin on head and neck squamous cell carcinoma (HNSCC) cell lines. The authors show that melatonin induces intracellular ROS, whose accumulation plays a role in mitochondrion-mediated apoptosis and autophagy though upstream modulation. These findings indicate that melatonin, when combined with cisplatin and radiotherapy, is a potential adjuvant agent. In another study by Y. Lin et al., cucurbitacin B has been identified as a compound with antiaging activity in yeast *Saccharomyces cerevisiae* through regulating autophagy, ROS, antioxidant ability, and aging-related genes.

Resistance to tumor necrosis factor-related apoptosis-inducing ligand (TRAIL) in cancer cells is a huge obstacle to creating effective TRAIL-targeted clinical therapies. A study by L. Hu et al. shows that SNX-2112, an Hsp90 inhibitor, when combined with TRAIL treatment synergistically enhanced TRAIL-induced cytotoxicity in HeLa cells. They found that SNX-2112 downregulated antiapoptotic proteins, inhibited AKT/mTOR signaling, and induced autophagic cell death upon the activation of a ROS/JNK/p53 signaling axis. The findings reported by the authors may provide a novel strategy to overcome apoptosis resistance during cancer treatment.

Contrast-induced nephropathy (CIN) is a leading cause of hospital-acquired acute kidney injury (AKI), but its physiopathology and therapeutic targets remain poorly characterized. X. Gong et al. now report that this morbid condition courses with the impairment of mitochondria quality control and induction of CCL2/CCR2-mediated inflammation, pointing towards novel mechanisms by which tetramethylpyrazine (TMP) prevents CM-induced kidney injury.

M. Cordani et al. summarize the current knowledge on sestrins (SESNs), a family of stress surveillance proteins which play a key role in the integration of ROS control and autophagy regulation in cancer- and age-related disorders and may constitute an interesting source of novel therapeutic opportunities. Hepatocellular carcinoma (HCC) is an aggressive tumor with a very poor prognosis for which several environmental risk factors, particularly viral infections and alcohol abuse, have been shown to promote carcinogenesis via augmentation of oxidative stress and autophagy. F. Ciccarone et al. review in depth our current understanding of the interplay between ROS and autophagy in HCC and their links to risk factors, environmental stress conditions, and therapeutic treatments.

Mitophagy is an essential cellular process that involves the selective degradation of dysfunctional and/or damaged mitochondria by autophagy and is requested for mitochondrion turnover and for responding to novel energetic requirements. In another review article, E. Vernucci et al. discuss the dual role that mitophagy plays in cancer- and age-related pathologies, as a consequence of oxidative stress, focusing on mechanisms and molecular targets for its therapeutic control using nanoparticles. Poly(ADP-ribosyl)ation (PARylation) is a covalent and reversible posttranslational modification (PTM) of proteins mediated by poly(ADP-ribose) polymerases (PARPs) with functions in DNA repair, replication, genome integrity, cell cycle, and metabolism. J. M. Rodriguez-Vargas et al. review the current understanding of PARP1 activation and PARylation in response to starvation-induced autophagy.

Some DNA damage sensors, such as FOXO3a, ATM, ATR, and p53, are important autophagy regulators, and autophagy seems therefore to have a role in DNA damage response (DDR). The existence of a link between autophagy and DDR is corroborated by the evidence that alterations in autophagy lead to increased DNA damage, highlighting its fundamental role in the maintenance of genomic stability. In this regard, S. Galati et al. provide novel insights about the role of autophagy in cell response to genotoxic stress. They report that the modulation of autophagy is a successful approach to reduce toxicity or to enhance the activity of anticancer drugs.

Epithelial mesenchymal transition (EMT) is considered a driving force in tumor progression, and increasing evidence reveals that ROS are crucial players in EMT engagement. In this sense, Y. Sun et al. report that activating ferritinophagic flux, by a novel iron chelator, leads the enhancement in ROS production, highlighting its relevance as a driving force in EMT fate.

In summary, the broad coverage in this special issue provides several new perspectives on the biology of ROS and autophagy regulation with an emphasis on molecular aspects and novel therapeutic approaches and places the interplay between autophagy regulation and oxidative stress as a priority subject in biomedical research addressing aging and cancer biology.
